# Effects of Spatial Memory Processing on Hippocampal Ripples

**DOI:** 10.3389/fneur.2021.620670

**Published:** 2021-03-05

**Authors:** Daniel Lachner-Piza, Lukas Kunz, Armin Brandt, Matthias Dümpelmann, Aljoscha Thomschewski, Andreas Schulze-Bonhage

**Affiliations:** ^1^Epilepsy Center, Medical Center–University of Freiburg, Faculty of Medicine, University of Freiburg, Freiburg im Breisgau, Germany; ^2^Spemann Graduate School of Biology and Medicine (SGBM), University of Freiburg, Freiburg im Breisgau, Germany; ^3^Faculty of Biology, University of Freiburg, Freiburg im Breisgau, Germany; ^4^Department of Neurology, Paracelsus Medical University Salzburg, Salzburg, Austria

**Keywords:** high-frequency oscillations, ripples, interictal epileptiform spikes, sleep spindles, hippocampus, cognition, memory consolidation, spatial memory

## Abstract

Human High-Frequency-Oscillations (HFO) in the ripple band are oscillatory brain activity in the frequency range between 80 and 250 Hz. HFOs may comprise different subgroups that either play a role in physiologic or pathologic brain functions. An exact differentiation between physiologic and pathologic HFOs would help elucidate their relevance for cognitive and epileptogenic brain mechanisms, but the criteria for differentiating between physiologic and pathologic HFOs remain controversial. In particular, the separation of pathologic HFOs from physiologic HFOs could improve the identification of epileptogenic brain regions during the pre-surgical evaluation of epilepsy patients. In this study, we performed intracranial electroencephalography recordings from the hippocampus of epilepsy patients before, during, and after the patients completed a spatial navigation task. We isolated hippocampal ripples from the recordings and categorized the ripples into the putative pathologic group *iesRipples*, when they coincided with interictal spikes, and the putative physiologic group *isolRipples*, when they did not coincide with interictal spikes. We found that the occurrence of *isolRipples* significantly decreased during the task as compared to periods before and after the task. The rate of *iesRipples* was not modulated by the task. In patients who completed the spatial navigation task on two consecutive days, we furthermore examined the occurrence of ripples in the intervening night. We found that the rate of ripples that coincided with sleep spindles and were therefore putatively physiologic correlated with the performance improvement on the spatial navigation task, whereas the rate of all ripples did not show this relationship. Together, our results suggest that the differentiation of HFOs into putative physiologic and pathologic subgroups may help identify their role for spatial memory and memory consolidation processes. Conversely, excluding putative physiologic HFOs from putative pathologic HFOs may improve the HFO-based identification of epileptogenic brain regions in future studies.

## Introduction

High Frequency Oscillations (HFO) are an electrographic manifestation of hyper-synchronized neurons and are subdivided into Ripples and Fast-Ripples according to the frequency range of the oscillations (80–250 and 250–500 Hz, respectively) (1, 2). In the field of epilepsy, Ripples and Fast-Ripples were initially considered improved biomarkers of epileptogenic networks (3–8). However, recent research has drawn a more complex picture (9–11) and has highlighted the importance of being able to differentiate between physiologic and pathologic HFOs.

Interictal Epileptic Spikes (IES) are another type of epileptic biomarker. IES are common in patients with epilepsy, have a waveform of a fast transient, are commonly generated in epileptic cortex and reflect a hyper-excitability of neural networks (12). IES are very sensitive but not specific to epileptogenic areas (13, 14). Ripples are known to coincide temporally and spatially with IES to some extent. These IES coincident Ripples appear to have different amplitude and waveform characteristics when compared to Ripples associated with physiologic events such as sleep spindles (15). IES coincident Ripples may be more sensitive to the seizure-onset-zone than Fast-Ripples and also more specific to it than Ripples occurring in isolation from IES (16). IES coincident Ripples may better predict post-surgical outcomes than Ripples not coinciding with IES (17) and Ripples coinciding with IES showed the highest correspondence with the resected volume in seizure-free patients as compared to other HFO subgroups (18). Moreover, a combined marker composed of IES and HFO occurrence rates appeared to be useful for estimating the epileptogenic zone (11). Together, the coincidence with IES may constitute a good criterion for separating pathologic Ripples from physiologic Ripples.

Sleep spindles are a third type of electrographic pattern which is observed in the human electroencephalogram (EEG) recorded with scalp or implanted electrodes (19). Sleep-spindle events have a distinct oscillatory waveform with durations between 0.5 and 3.0 s and frequencies between 11 and 16 Hz (20–22). Sleep spindles are generated and controlled by thalamic networks, with several hypotheses linking them to gating functions of sensory information flow. However, so far, the complete and definitive functional meaning of sleep spindles remains to be explored (23). Amongst others, sleep spindles may be relevant for memory consolidation during sleep, particularly when coupled to hippocampal Ripples (24–32). The occurrence rate of sleep spindle-coupled Ripples during sleep may thus reflect the intensity of memory consolidation.

Based on this prior knowledge, we hypothesized in the current study that hippocampal ripples could be differentiated into a putatively pathologic subgroup (*iesRipples*) and a putatively physiologic subgroup (*isolRipples*) based on their temporal and spatial coincidence with IES: *iesRipples* should coincide with an IES temporally (i.e., occurring within the duration of an IES) and spatially (i.e., when recorded on the same channel as an IES), whereas *isolRipples* should occur in isolation. Moreover, we hypothesized that ripples temporally co-occurring with ipsilateral hippocampal sleep spindles (*spindleRipples*) could serve as a marker for memory-consolidation processes. In our analyses, we therefore assessed whether the occurrence rates of *iesRipples* and of *isolRipples* were altered during the spatial navigation task. The spatial navigation task required the patients to form associative memories between objects and their corresponding locations and thus imposed an increased cognitive demand on the patients. Since physiological ripples are associated with cognitive functioning, we hypothesized that the rate of *isolRipples* should be altered during the task, whereas the activity of *iesRipples* should be unaffected. Additionally, in patients who performed the spatial navigation task on two consecutive days, we assessed the correlation between the occurrence rate of *spindleRipples* in the intervening night and the performance improvement of the patients between both days. Based on the proposed role of sleep spindle-coupled ripples in memory consolidation, we hypothesized that a higher rate of *spindleRipples* should be associated with a greater performance improvement.

## Methods

### Patient Selection

Participating patients (Table 1) suffered from pharmaco-resistant focal epilepsy and underwent intracranial EEG (iEEG) recordings from the hippocampus to identify their seizure onset zone at the Freiburg Epilepsy Center, Germany. The clinical decision for the implantation of iEEG electrodes was made individually for each patient in cases when the epileptogenic zone remained unclear using non-invasive methods. All patients gave their informed consent to participate in a study aiming at the identification of electrophysiological correlates of cognitive processing, including a spatial navigation task. A total of 19 patients performing the spatial navigation task were included in the current study, 9 of which completed the spatial navigation task on two subsequent days. All patients gave their written informed consent and the study was approved by the Ethics Committee of the Freiburg University Medical Center.

**Table 1:** Patient information.

**Patient ID**	**Age**	**Sex**	**Paradigm No**.	**No. trials**	**Duration pre-, intra- and post-paradigm phases (m)**	**No. hippocampal bipolar channels**	**Implantation side**
1	45	F	1	51	48	4	Bilateral
2	34	M	1	129	51	3	Right
			2	152	45		
3	28	F	1	160	51	3	Right
			2	131	53		
4	36	F	1	91	53	1	Right
			2	85	39		
5	28	M	1	160	47	2	Left
6	29	M	1	160	54	4	Bilateral
			2	160	48		
7	44	F	1	54	21	5	Bilateral
			2	60	43		
8	23	F	1	98	41	4	Bilateral
9	25	F	1	151	72	2	Left
			2	81	39		
10	27	F	1	76	36	2	Left
11	32	M	1	91	57	3	Right
			2	114	45		
12	48	M	1	126	64	1	Left
13	49	M	1	160	81	4	Bilateral
14	25	F	1	82	46	3	Right
15	29	M	1	102	42	1	Right
16	49	M	1	54	39	4	Bilateral
17	43	M	1	102	57	1	Left
18	27	F	1	31	18	1	Right
			2	75	39		
19	19	M	1	160	58	4	Bilateral
			2	160	50		

### Spatial Navigation Paradigm

**Figure 1:**
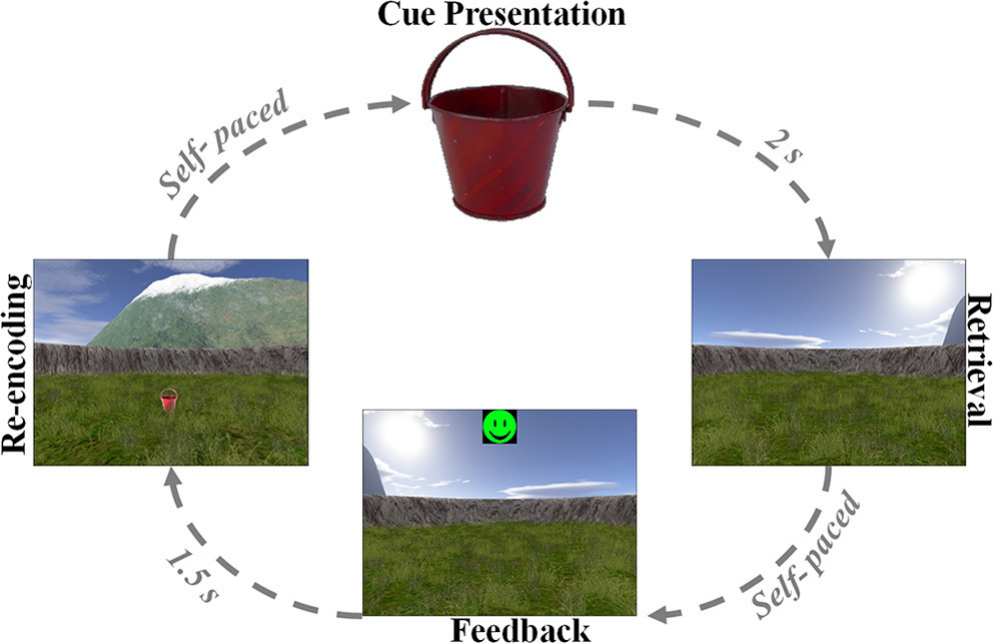
Associative object-location memory task during virtual spatial navigation. At the beginning of the experiment, patients collected eight different objects from different locations within the virtual environment. Afterward, patients completed variable numbers of retrieval trials, during which they were first presented with one of the eight objects serving as cue (cue presentation). Patients then navigated to the remembered location of that object (retrieval) and made a response. Following this response, patients received feedback *via* an emoticon (feedback) and had to collect the object from its correct location (re-encoding).

The spatial navigation paradigm was adapted from previous studies (33–35). In this paradigm, the patients performed an object-location memory task navigating freely in a circular virtual environment. The environment comprised a grassy plain bounded by a cylindrical cliff. Two mountains, a sun, and several clouds provided patients with distal orientation cues. Patients completed the task on a laptop using the arrow keys for moving forward and turning left and right and the spacebar to indicate their response. Patients were asked to complete up to 160 trials but were instructed to pause or quit the task whenever they wanted. At the very beginning, patients collected eight everyday objects (randomly drawn from a total number of 12 potential objects) from different locations in the arena. Objects appeared one after the other. Afterward, patients completed variable numbers of trials, depending on compliance. Each trial consisted of four different phases (Figure 1). First, one of the eight objects was presented for 2 s (cue presentation). Afterwards, patients were asked to navigate to the associated object location within the virtual environment (retrieval). After patients had indicated their response via a button press at the assumed object location, they received feedback depending on response accuracy. Response accuracy was measured as the distance between the remembered location and the correct location (drop error). Last, the object was presented in the correct location, and patients had to collect the object from there to further improve their associative memory between the object and its location. After each trial, a fixation crosshair was shown for a variable duration of 3–5 s. Triggers were detected using a phototransistor attached to the screen marking onsets and offsets of the cue presentation phase. The intra-paradigm period of the iEEG was then delimited by the first and last phototransistor triggers. We calculated the patients' performance in each trial as the ratio between the drop error and the largest possible drop error (maximum random drop error):

$$\begin{array}{l} Performance_{Single\;Trial}=1-\;\frac{Drop\;Error}{Maximum\;Random\;Drop\;Error} \end{array}$$

The largest possible drop error in a given trial was determined by creating one million random locations within the virtual environment and then selecting the location with the largest Euclidean distance to the correct object location. The performance for the entire paradigm was calculated as the median performance across all trials.

### Identification of Hippocampal and White Matter Channels

Preimplantation and post-implantation MRI scans were available for all patients. Electrode localization was performed using FSL (https://fsl.fmrib.ox.ac.uk/fsl/fslwiki/FSL) and PyLocator (http://pylocator.thorstenkranz.de/). The post-implantation MR image was coregistered with the preimplantation MR image. Next, the preimplantation MR image was skull-stripped and normalized to MNI space, and the same normalization matrix was applied to the post-implantation MR image. Normalized post-implantation images were visually inspected using PyLocator, and channel locations were manually identified. For the analyses in this study, we only considered electrode channels located in the right or left hippocampus.

### Detection of Ripples, *isolRipples, iesRipples*, and IES

Intracranial EEG data were recorded using “Profusion EEG Software” (Compumedics Limited, Abbotsford Victoria, Australia). Original signals were low-pass filtered at 800 Hz and sampled at 2 kHz using Cz as a hardware reference. All signal analyses were performed using bipolar montages.

The detection of Ripples and IES on the hippocampal iEEG signals was accomplished by using automatic detectors (36), which were based on multivariate classifications of iEEG epochs using kernelized support-vector-machines. The spectral analysis of these detections was performed using the Morlet wavelet transform, the maximum and minimum frequencies clustering the events' power corresponded to those frequency-bins with a power contribution within that one of the spectral peak (i.e., the frequency with the maximum power contribution) +/– the standard deviation of the power across all frequency bins.

The automatic detectors were run on time-selected segments of the hippocampal iEEG signals. These segments corresponded to the pre-, intra- and post-paradigm phases. The intra-paradigm phase was delimited by the first and last cue-presentation triggers. The duration of the pre- and post-paradigm phases was the same as for the intra-paradigm phase. The pre-paradigm phase ended 30 min before the start of the intra-paradigm phase and the post-paradigm phase started 30 min after the end of the intra-paradigm phase.

**Figure 2:**
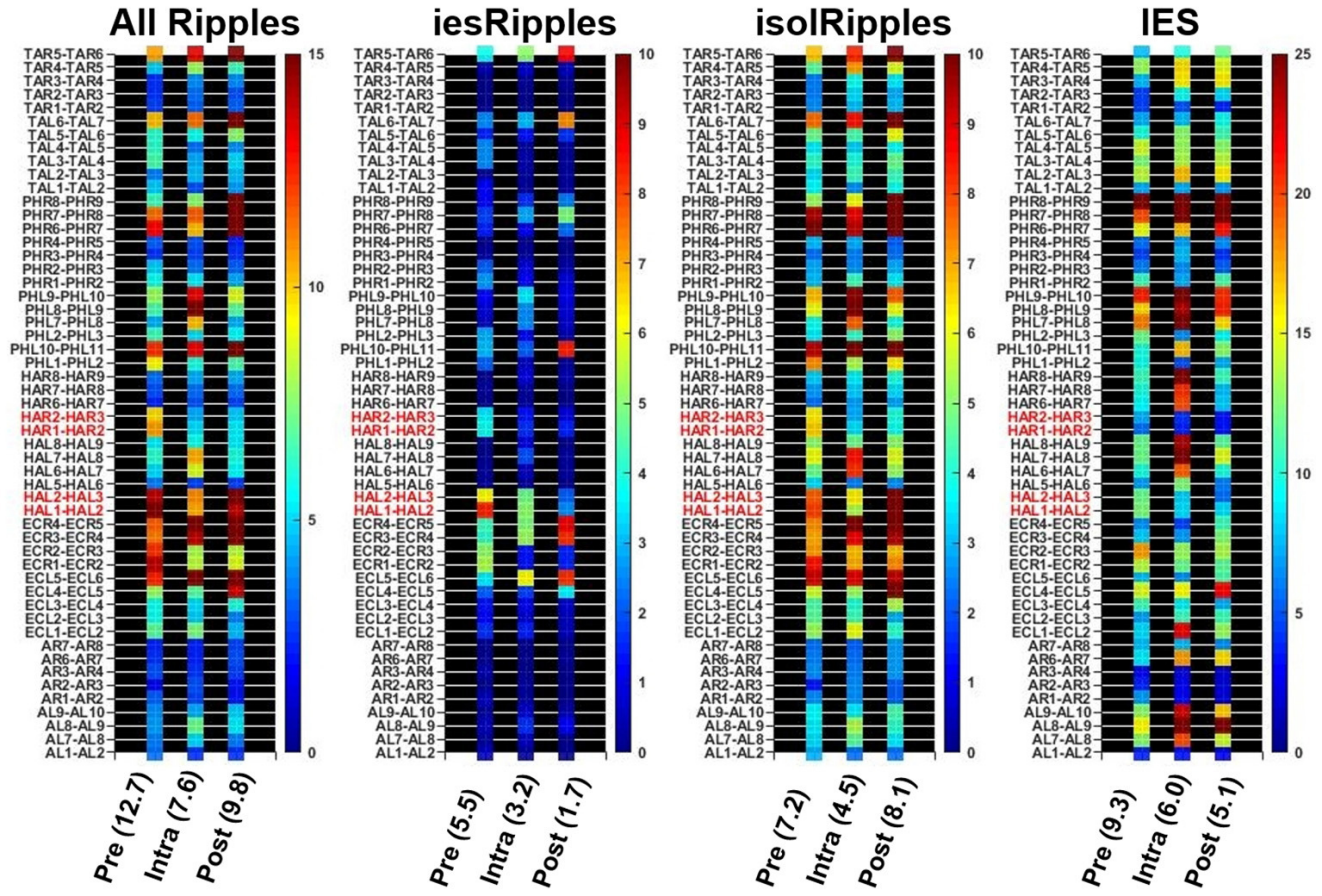
Determining the hippocampal activity for Patient 16 and each event class. Firstly, the automatic detections of Ripples, *iesRipples, isolRipples*, and IES were used to characterize each channel (y-axes) with their occurrence rate per minute (heat-maps). The specific brain region Hippocampus (red channel labels) was then characterized by the average occurrence rate across all hippocampal channels. The average hippocampal activity for each event class and the pre-, intra- and post-paradigm segments is shown in parentheses on the x-axis.

The automatic detectors provided discrete events of the classes Ripples and IES. Each event comprised a start time and an end time. We used custom scripts in MATLAB 2018b that determined the temporal and spatial coincidence of the Ripples and IES in order to identify ripples belonging to the class *iesRipples*. Ripples which were not coincident with IES formed the class *isolRipples*. For each class of ripples, we then calculated their occurrence rate per minute in each of the hippocampal channels. If a patient had more than one hippocampal electrode, we averaged the occurrence rates across the different hippocampal channels (Figure 2).

### Analysis of the Ripples, *isolRipples, iesRipples*, and IES Activity

We tested for the effect of the spatial navigation paradigm on the occurrence rate of Ripples, *isolRipples, iesRipples*, and IES using the data from all 19 patients. In patients who performed the task on two consecutive days, we only used the data from the first day for this analysis in order to avoid that these patients had a stronger effect on the statistical results. A mixed ANOVA was conducted to test for main effects and interactions between the factors “time period” (pre-, intra- and post-paradigm phase) and “ripple class” (*isolRipples* vs. *iesRipples*) on the occurrence rate of hippocampal ripples. In a *post-hoc* analysis, we firstly used a two-tailed, non-parametric Wilcoxon signed rank test to analyze whether the occurrence rates differed (i) between the pre-paradigm and the intra-paradigm phase, (ii) between the intra-paradigm phase and the post-paradigm phase, and (iii) between the control phases pre-paradigm and post-paradigm; at this stage no correction for multiple comparisons was applied since the families (i.e., pre vs. intra, intra vs. post, pre vs. post) had no repeated analyses (i.e., α = 0.05) (37). Finally, as part of the same *post-hoc* analysis, we performed either a subsequent left-tailed or a right-tailed Wilcoxon signed rank test to analyze if the activity-difference between phases corresponded to an increase or a decrease; at this stage a Bonferroni correction was applied to the significance threshold (i.e., α = 0.025) since two null hypotheses were considered (two-tailed and one-tailed Wilcoxon signed rank tests).

### Detection of *spindleRipples*

The *spindleRipple* events corresponded to those Ripples which were temporally and spatially coincident with hippocampal sleep spindles. The detection of hippocampal sleep spindles was achieved using an automatic detector (38) based on multivariate classifications of iEEG epochs using kernelized support-vector-machines. We used a custom MATLAB 2018b script to determine coinciding ripples and sleep spindles, which then composed the class *spindleRipples*. Only ripples that were completely within the start and end time of a sleep spindle were considered to be temporally coincident. The spatial coincidence of a ripple and a sleep spindle was present if they were both hippocampal and ipsilateral. All ripples complying with these rules of temporal and spatial coincidence comprised the *spindleRipples* class.

We estimated the occurrence rate of *spindleRipples* in patients who performed the spatial navigation task on two consecutive days. For all these patients, we used the data between 10:00 pm of the first day and 6:00 am of the second day to estimate the occurrence rate of *spindleRipples*. This time period was selected with the aim of maximizing the inclusion of non-rapid eye movement N2 sleep stages, since the occurrence and power of sleep spindles is highest during this sleep stage (21, 23, 39, 40). If a patient had more than one hippocampal electrode channel, we averaged the channel-specific *spindleRipple* rates across the different channels to obtain one overall occurrence rate, which quantified the number of *spindleRipples* per minute.

### Analysis of *spindleRipples* and Their Correlation With Spatial Navigation Performance

This analysis was only performed with the data from the patients who performed the spatial navigation task on two consecutive days. To test whether the occurrence rates of *spindleRipples* could be a marker of memory consolidation of the associative object-location memories that the patients formed during the spatial navigation task, we calculated the correlation between *spindleRipple* rates and the difference in performance obtained on days 1 and 2 (performance Δ). A non-paired, non-parametric, left-tailed Mann–Whitney U test was applied to each patient using all their trials from days 1 and 2 to test if the performance Δ was significant. The correlation between the hippocampal *spindleRipple* rates and the performance Δ was measured using Spearman's rank correlation coefficient. As a control, we quantified this relationship while controlling for the potential effect of the number of trials on day 1 using a partial correlation.

## Results

### Navigation Paradigm

**Figure 3:**
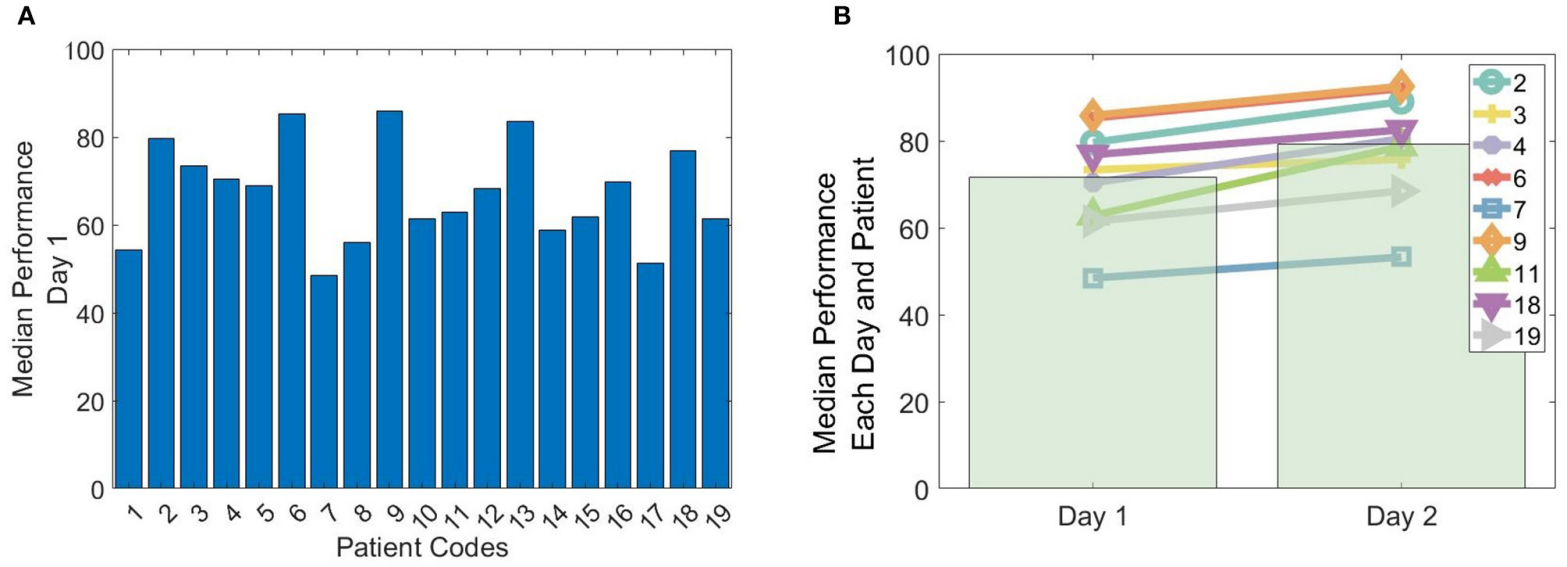
Performance in the spatial navigation paradigm. **(A)** Median performance across trials obtained by the 19 patients on their first (or only) day of conducting the spatial-navigation task. **(B)** The line plots depict the median performance across trials for each of the nine patients that conducted the spatial navigation paradigm on 2 consecutive days (day 1 and 2). The bar plots depict the average across patients.

A total of 19 patients completed the spatial navigation task (day 1). A subgroup of nine patients completed the spatial navigation task also on the subsequent day (day 2). The performance from each patient was measured as the median performance across all trials. For day 1, the maximum, minimum and mean performance corresponded to 86% (patient 9), 48% (patient 7) and 67 ± 11% (mean ± *SD*), respectively (Figure 3A).

In the subgroup of the 9 patients conducting the paradigm on two consecutive days, the maximum, minimum and mean performance on day 1 corresponded to 86% (patient 9), 48% (patient 7), and 72 ± 12%, respectively. On day 2, the maximum, minimum, and mean performance corresponded to 93% (patient 9), 53% (patient 7), and 79 ± 13%, respectively (Figure 3B). All patients showed a significant performance improvement, when comparing day 1 with day 2 (left-tailed Mann–Whitney *U*, all *p* < 0.025).

### Detection of *isolRipples* and *iesRipples*

**Figure 4:**
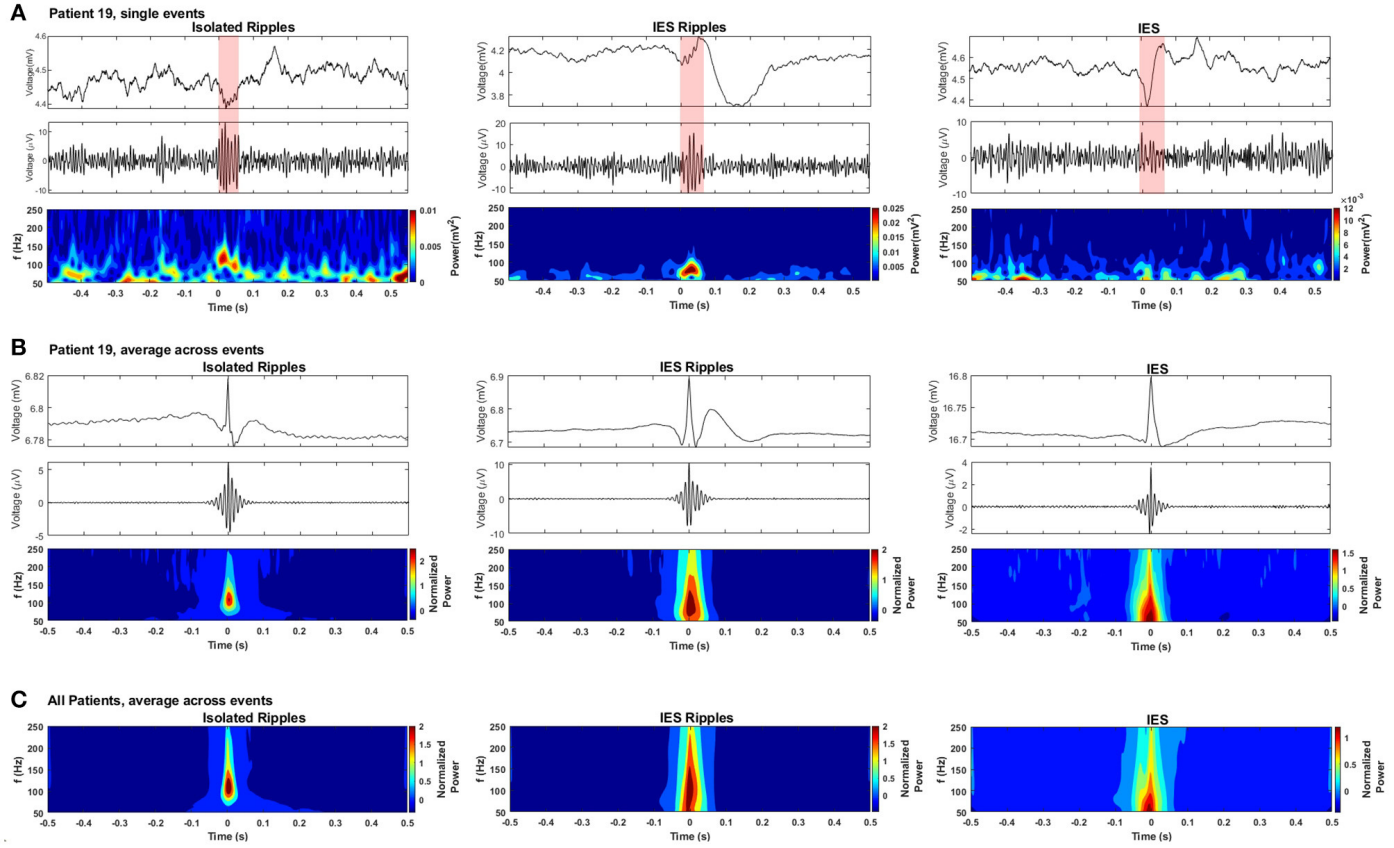
Detected hippocampal ripples and spikes. **(A)** Selected events from each event type: the- first row shows the waveform from the raw EEG, the second row shows the waveform from the bandpassed EEG (80–250 Hz), the third row shows the spectrogram from each event-type obtained using a Morlet wavelet transform. **(B)** Average event-types from patient 19. **(C)** Grand average of the three event-types from all patients.

The automatic detection on the hippocampal channels generated an average of 1,762 Ripple events and 1,042 IES events per patient across the pre-, intra- and post-paradigm phases. Of these Ripples, 74% occurred isolated from IES and were thus labeled *isolRipples*; the remaining 26% of the Ripples occurred spatially and temporally coinciding with an IES and were thus labeled *iesRipples*. A depiction of example waveforms and the corresponding spectrograms of *isolRipples* and *iesRipples* is provided in Figure 4.

A challenge when detecting HFOs is the correct handling of fast transients which, when filtered, can produce artifacts resembling authentic HFOs (41, 42). A depiction of the waveform and spectrogram of the IES detections is provided in Figure 4 in order to show the handling of fast transients by the automatic detectors and to allow the comparison with the spectrogram from the detected Ripples. The *isolRipples* showed a distinct increase in power within a narrow band which resembled a blob in the spectrogram, the tuples (Hz) consisting of the spectral-peak, blob-lower-frequency and blob-higher-frequency for the single-event, averaged-events and patients-average were (117, 82, 144), (109, 82, 144), and (109, 88, 144), respectively. This blob-like power increase was also shown by *iesRipples* when considering only patient 19. The same tuples for *iesRipples* were (82, 82, 102), (102, 82, 144), and (109, 82, 165), respectively.

The spectrogram of the IES depicted in the third column of Figure 4 showed typical spectral characteristics of a fast-transient, which much like a single pulse are represented in all frequency bins when analyzed by a time-frequency transformation. The key characteristic that differentiates a filtering artifact from a real HFO event is then this blob-like, narrow-band power increase which is shown by the spectrograms of both *isolRipples* and *iesRipples*.

The events forming the averages in Figure 4 were centered using their maximum peak. Hence a peak was formed at the center of the raw averages while surrounding samples were canceled because of varying pre- and post-event waveforms across events.

### Modulation of Ripple Activity by Increased Cognitive Demands During the Spatial Navigation Task

The mixed ANOVA test showed a non-significant main effect of time period (pre-, intra-, post-paradigm) (*F* = 0.679, *p* = 0.510), a significant main effect of the ripple class (*isolRipples, iesRipples)* (*F* = 8.948, *p* < 0.005), and a significant interaction between both factors (*F* = 4.069, *p* < 0.025). To further understand this interaction, we performed *post-hoc* analyses (Wilcoxon signed rank tests).

We found no significant differences in the occurrence rates of *allRipples* for the pair-wise comparisons (pre-paradigm vs. intra-paradigm, *p* < 0.717; intra-paradigm vs. post-paradigm, *p* < 0.243; pre-paradigm vs. post-paradigm, *p* < 0.314). Hence, the increased cognitive load exerted by the spatial-navigation paradigm did not modulate the activity from the *allRipples* event class.

**Figure 5:**
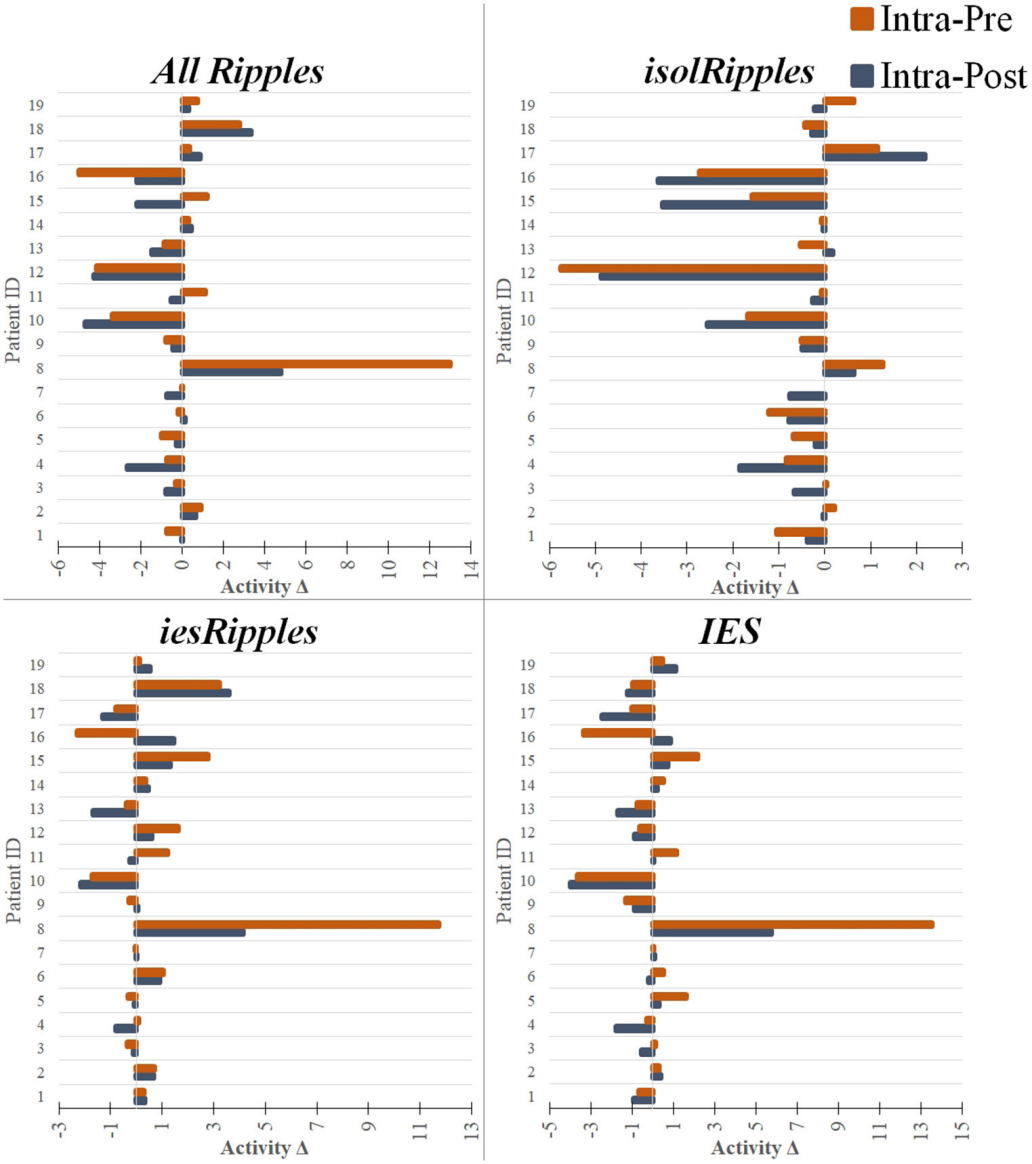
Modulation of the event-classes by the increased cognitive load. The bar plots show pairs of activity differences between phases (Activity Δ), i.e., difference between the intra- and pre-paradigm phase (orange) and difference between the intra- and post-paradigm phase (blue), for each patient and using each of the event-classes. The bar plots with a negative value indicate a decrease in activity when entering or leaving the period of increased cognitive load (i.e., intra-paradigm phase). The bar plots with a positive value indicate an increase in activity during the paradigm when compared to the pre- or post-paradigm phases. The hippocampal *isolRipples* were the only event-class to show a significant modulation by the navigation task, presenting an activity decrease when comparing the intra-paradigm occurrence rates with those from both the preceding (pre-paradigm, *p* < 0.043) and succeeding (post-paradigm, *p* < 0.007) control phases.

For *isolRipples*, the comparison pre-paradigm vs. intra-paradigm showed a significant difference (*p* < 0.043), the subsequent right-tailed test showed a significance of *p* < 0.022, hence the *isolRipples* presented an activity decrease when transitioning from the pre- to the intra-paradigm phase. Similarly, the comparison of *isolRipples* from the intra-paradigm vs. post-paradigm phase showed a significant difference (*p* < 0.007), the subsequent left-tailed test showed a significance of *p* < 0.004. Hence, the *isolRipples* presented an activity increase when transitioning from the intra- to the post-paradigm phase. When comparing the occurrence rates of *isolRipples* during the control phases pre-paradigm and post-paradigm, no significant difference was found (*p* > 0.314). In summary, the increased cognitive load exerted by the spatial navigation paradigm did modulate the activity from the *isolRipples* in a way that their activity was reduced during the paradigm (Figure 5), additionally, the *isolRipples* activity returned to the pre-paradigm control values after the phase of increased cognitive load.

We found no significant differences in the occurrence rates of *iesRipples* for the pair-wise comparisons (pre-paradigm vs. intra-paradigm, *p* > 0.277; intra-paradigm vs. post-paradigm, *p* > 0.295; pre-paradigm vs. post-paradigm, *p* > 0.260). Thus, the increased cognitive load exerted by the spatial navigation paradigm did not modulate the activity from the *iesRipples* event class.

For IES, we found no significant differences for the pair-wise comparisons pre-paradigm vs. intra-paradigm (*p* > 0.778) and intra-paradigm vs. post-paradigm phase (*p* > 0.260); however, the IES activity from the control phase pre-paradigm was significantly different from the post-paradigm activity (*p* < 0.022), the subsequent left-tailed test showed a significance of *p* < 0.012. These results suggest that the IES activity was not modulated by the increased cognitive load, however the IES activity from the post-paradigm control phase was higher than the IES activity during the pre-paradigm control phase.

The results described in this section are also shown in Table 2 and summarized in Figure 5.

**Table 2:** Ripple and IES activity in the pre-, intra- and post-paradigm iEEG segments.

**Patient ID**	**iEEG segment duration (min)**	**Average hippocampal occurrence rate / min**											
		**All Ripples**			***isolRipples***			***iesRipples***			***IES***		
		**Pre**	**Intra**	**Post**	**Pre**	**Intra**	**Post**	**Pre**	**Intra**	**Post**	**Pre**	**Intra**	**Post**
1	48	9.06	8.30	8.34	7.21	6.16	6.55	1.85	2.13	1.80	4.80	4.14	5.05
2	51	2.82	3.73	3.09	2.03	2.24	2.27	0.79	1.49	0.82	2.85	3.17	2.78
3	51	7.81	7.50	8.30	6.82	6.86	7.53	0.99	0.64	0.77	3.32	3.48	4.04
4	53	7.54	6.77	9.41	4.52	3.68	5.53	3.02	3.09	3.88	3.99	3.69	5.46
5	47	15.02	14.02	14.30	13.12	12.43	12.63	1.90	1.59	1.67	6.67	8.29	7.98
6	54	8.04	7.86	7.73	5.00	3.78	4.55	3.04	4.08	3.17	4.42	4.93	5.14
7	21	9.74	9.70	10.43	9.40	9.40	10.16	0.34	0.30	0.27	5.81	5.84	5.74
8	41	12.81	25.79	21.01	4.58	5.85	5.21	8.23	19.94	15.79	11.84	25.37	19.60
9	72	11.64	10.85	11.28	10.80	10.28	10.78	0.84	0.56	0.49	5.92	4.61	5.47
10	36	11.04	7.65	12.37	6.21	4.54	7.09	4.83	3.11	5.28	14.17	10.51	14.49
11	57	7.29	8.43	8.95	5.07	4.99	5.26	2.22	3.43	3.69	3.61	4.77	4.71
12	64	18.11	13.97	18.24	16.25	10.48	15.37	1.87	3.48	2.87	5.02	4.41	5.27
13	81	16.41	15.51	17.00	15.57	15.05	14.88	0.84	0.46	2.12	6.95	6.19	7.88
14	46	7.41	7.71	7.27	4.66	4.58	4.61	2.75	3.13	2.66	8.14	8.68	8.46
15	42	20.81	22.01	24.22	17.54	15.95	19.48	3.27	6.06	4.73	7.36	9.56	8.83
16	39	12.66	7.65	9.83	7.19	4.46	8.09	5.46	3.19	1.74	9.29	5.96	5.11
17	57	15.71	16.08	15.19	14.43	15.58	13.39	1.27	0.49	1.80	6.98	5.99	8.43
18	18	21.05	23.82	20.49	3.00	2.55	2.83	18.05	21.27	17.66	31.15	30.21	31.43
19	58	10.27	11.03	10.70	8.58	9.23	9.45	1.69	1.80	1.26	5.99	6.49	5.39
Avg	49.3	11.85	12.02	12.53	8.53	7.80	8.72	3.33	4.22	3.81	7.80	8.23	8.49
Std.Dev.	14.73	4.80	6.03	5.38	4.63	4.34	4.57	3.95	5.81	4.64	6.17	7.05	6.62

### Improvement in Performance and the Correlation With *spindleRipples*

**Figure 6:**
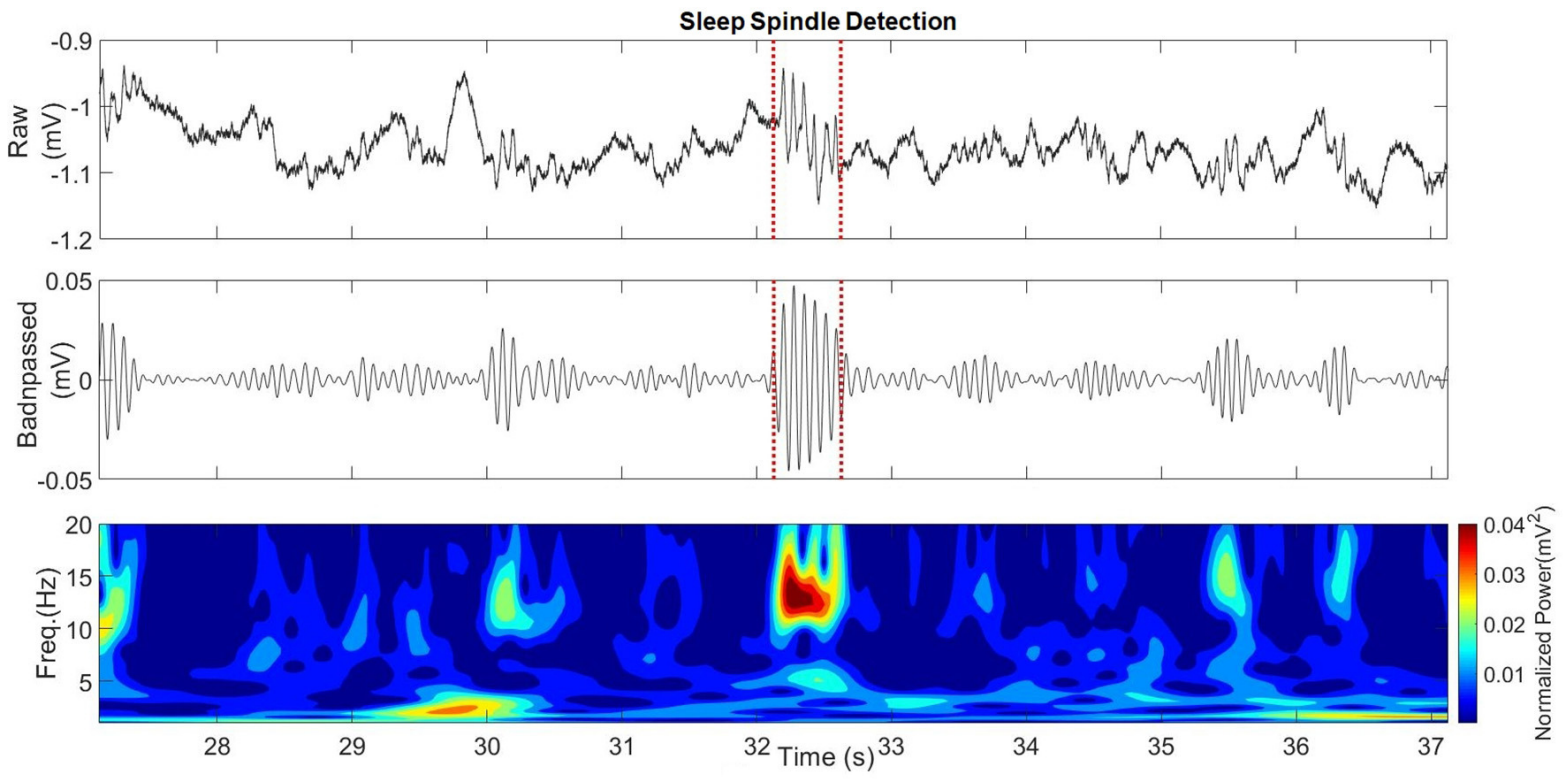
Hippocampal sleep spindle from Patient 4.

An example of a detected sleep spindle is presented in Figure 6. The performances obtained by the patients when conducting the spatial navigation task on days 1 and 2 are presented in Table 3.

**Table 3:** Performance improvement and hippocampal Ripple and *spindleRipple* activity.

**Patient code**	**Perf. day 1**	**Perf. day 2**	**Performance Δ**	**Improvement (Mann-Whitney *U*, *p*-value)**	**All Ripples (Occ. Rate/min)**	***spindleRipples* (Occ. Rate/min)**	**No. Trials day 1**	**No. Trials day 2**
2	79.6	89	9.4	4.9 × 10^−21^	7.19	0.70	129	151
3	73.3	75.7	2.4	9.1 × 10^−3^	8.3	0.36	160	130
4	70.4	80.5	10.1	4.9 × 10^−10^	13.86	1.00	90	84
6	85.3	92	6.7	4.8 × 10^−20^	14.75	0.75	160	160
7	48.4	53.3	4.9	1.3 × 10^−2^	12.16	0.64	53	59
9	85.9	92.5	6.6	7.2 × 10^−15^	14.67	1.83	150	80
11	62.8	78.7	15.9	8.0 × 10^−15^	15.74	2.81	90	113
18	76.8	82.5	5.7	1.0 × 10^−2^	23.3	0.06	30	74
19	61.5	68.5	7	3.7 × 10^−6^	10.18	2.12	160	160

**Figure 7:**
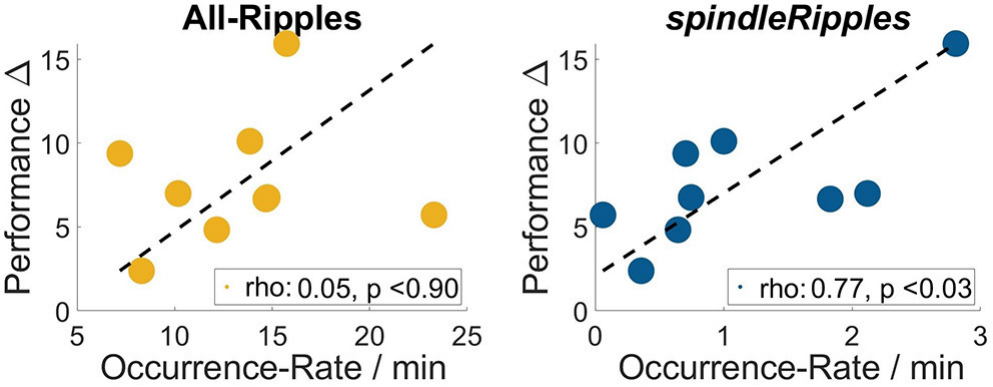
Occurrence Rates of the event-class all hippocampal ripples and *spindleRipples* and their correlation with the patients' difference in performance when comparing day 1 and 2 (i.e., performance Δ).

As previously mentioned, all the patients' performance for the navigation task improved on day 2 when comparing it with day 1 (left-tailed Mann–Whitney *U* all *p* < 0.025). During the night between the two sessions of the spatial navigation task, hippocampal Ripples were detected and their occurrence rate (incidences per minute) was obtained. We found that the activity from the *allRipples* event class showed no correlation with the patients' performance improvement when repeating the spatial navigation task (*rho* = 0.13, *p* = 0.74; controlling for the number trials on day 1: *rho* = 0.05, *p* = 0.90). In contrast, the occurrence rate of the detected *spindleRipples* showed a significant positive correlation with performance improvement (*rho* = 0.73, *p* = 0.03; controlling for the number of trials on day 1, *rho* = 0.77, *p* < 0.03) (Figure 7).

As a control, we also computed correlations between the occurrence rate of ripples and the performance values on both days in order to present evidence that the association was presumably due to the learning process and not simply related to the patients' general performance level. These correlations were not significant (for performance on day 1 and *allRipples* rate, *rho* = 0.17, *p* = 0.68; performance on day 1 and *spindleRipples* rate, *rho* = −0.14, *p* = 0.74; performance on day 2 and *allRipples* rate, *rho* = 0.32, *p* = 0.41; performance on day 2 and *spindleRipples* rate, *rho* = 0.05, *p* = 0.91).

## Discussion

In this study, we examined the activity of putatively physiologic and pathologic ripples in the human hippocampus during a spatial navigation task. We differentiated between the two groups of ripples by analyzing their coincidence with interictal spikes. We found that the rate of the putatively physiologic ripples, *isolRipples*, decreased during the task as compared to pre- and post-task phases, whereas the putatively pathologic ripples, *iesRipples*, did not show this effect. In addition, the ripples associated with hippocampal sleep spindles, *spindleRipples*, showed a positive correlation with the performance improvement of patients who completed the spatial navigation task on two consecutive days.

In summary, the increased cognitive demands from the spatial navigation task exerted differential effects on *iesRipples* and *isolRipples*; these results [in addition to previously published evidence showing *iesRipples* as the most and *isolRipples* as the least accurate estimator of the epileptogenic-zone (18)] hence provide further evidence to support the putatively physiologic and pathologic nature initially attributed to *isolRipples* and *iesRipples*. Furthermore, our study suggests that ripples associated with sleep spindles may constitute a marker of memory-consolidation processes.

Our analysis was focused on the hippocampus, as it plays a major role for both spatial navigation (24–30) and declarative memory formation (43–50) and is a candidate region for generating epileptic activity (51, 52) and is therefore often assessed for the decision making and planning for surgical epilepsy therapy.

Our first goal was to analyze the activity of putative physiologic and putative pathologic Ripples during periods when the cognitive load was higher than normal and then compare this activity with controls coming from periods with a lower demand on cognitive functioning. During these periods with a lower cognitive demand, all patients stayed in their hospital bed in the fairly quiet and stable environment of their room. It can thus be assumed that cognitive demands during these periods were indeed lower than during the spatial navigation task. Furthermore, all hippocampal activities reported corresponded to the average across the duration of the pre-, intra-, and post-paradigm phases; short increases of cognitive-load during the control phases would have been averaged out. In contrast, during the intra-paradigm phase it is conceivable that the reported average was derived from a period of a constant and increased cognitive-load.

The periods selected as controls before and after the paradigm showed no difference in the activity from either *allRipples, isolRipples*, or *iesRipples*, which in the case of the allRipples and iesRipples, as previously mentioned, evidences a lack of modulation exerted by the period of increased cognitive-load. In the case of *isolRipples* however, this equivalence between controls also shows that the isolRipples activity was modulated by the increased cognitive-load and then returned to the levels previous to the conducted navigation task.

### Differentiation of Pathologic and Physiologic Ripples

Numerous publications have reported, for both the human and non-human brain, on the occurrence of Ripples during wakefulness and while conducting cognitive tasks (43–50); despite this, to our knowledge, only the studies from (47) and those from the Brázdil1 group (48–50), have analyzed the effects of cognitive processes on the activity of putatively physiologic and putatively pathologic Ripples during the awake state and in humans. The study from (50) presents the largest patient cohort with 36 patients and will thus be considered for further discussion. This study explored if the effect of cognitive load in the form of different tasks (visual oddball, Go/NoGo, Ultimatum Game, Mismatch Negativity) on putative-pathologic ripples (i.e., ripples from epileptic hippocampi, hereinafter referenced as *pathoBrazRipples*) differed from the effect on putative-physiologic ripples (i.e., from non-epileptic hippocampi, hereinafter referred to as *physioBrazRipples*). Both *pathoBrazRipples* and *physioBrazRipples* were reported to show a significant activity reduction when transitioning from the pre- to the intra-paradigm phase, however this reduction was more significant for the *physioBrazRipples* than for the *pathoBrazRipples* when averaging the activity across the analyzed hippocampal channels.

Our results agree with (50) in that both *isolRipples* and *physioBrazRipples* showed a decrease in their activity during the intra-paradigm phase when compared to either the resting states pre-paradigm or post-paradigm. Our results disagree with (50) in that in contrast to the decrease in activity of *pathoBrazRipples* during the task, our *iesRipples* did not show any modulation exerted by the increased cognitive load. The differences between our results and those from (50) are likely due to the fact that neither approach is exhaustive, i.e., both approaches are likely to increase the proportion of physiologic to pathologic Ripples but it is still possible that this formed sub-groups of Ripples are not exclusively physiologic or pathologic.

Another recent study, (47), examined ripple-occurrence rates across two cognitive tasks and a resting state during wakefulness. This study detected ripples firstly in the time domain by thresholding the power in the ripple band, and secondly by only accepting those detections with spectral power bursts narrowed down to the ripple range. This procedure used for the removal of potential artifactual ripples will produce detected events with spectral characteristics resembling those from our *isolRipples* class (Figure 4). Interestingly, in agreement with our findings for *isolRipples*, the results from (47) showed their ripple occurrence-rate to be higher during the resting state than during the cognitive tasks.

### Modulation of IES Activity

Interactions between epileptic activity and cognition have been discussed for many years (53). We compare our results with those obtained by other studies analyzing IES activity changes during cognitive tasks. The studies from (54–56) give evidence that cognitive tasks and movements can change the properties of epileptogenic networks and thus the occurrence of IES, these studies however provide disagreeing conclusions on the activity patterns followed by the reported IES. The work from (54) reported a reduction of the spike rate during successful encoding while conducting a visual recognition memory task in amygdala, hippocampus, and temporal cortex. In agreement with the latter study, (55) showed a decrease of IES activity during movement in two patients with a focal cortical dysplasia in the pre- and/or post-central gyrus. The more recent study by (56) presented an increase of temporal lobe interictal spikes in the hippocampus during a spatial memory task and both in hippocampus and lateral temporal lobe during an episodic memory task.

In contrast with the mentioned conflicting studies, our results did not show any significant modulation, whether increasing or decreasing, of the IES activity during the period of increased cognitive load. We did find, however, a difference between the control phases pre-paradigm and post-paradigm. The difference in activity from our control periods then calls for a further exploration of the importance of selecting a control period, which can then allow the comparison of results between studies.

### Correlation of *spindleRipples* and Memory Consolidation

The fact that all patients improved their performance when repeating the spatial-navigation paradigm provides evidence that the used paradigm did in fact exert a cognitive load which lead to the learning of newly acquired information. Interestingly, we found a strong correlation between *spindleRipple* activity and performance improvement.

To our knowledge only one other study has analyzed Ripple-rates and their correlation with cognitive-performance. This study from (24) analyzed the rates of ripples in the hippocampus and rhinal cortex during a short nap of 1 h, a set of images was presented pre- and post-nap and then again at the control stage, where patients had to distinguish known from novel images. Their results showed firstly, that the ripple events were circumscribed to the frequency range between 80 and 120 Hz. Secondly, that the ripple rate in the hippocampus was on average 1.90/min. Thirdly, that only rhinal, but not hippocampal ripples were correlated with the number of correctly recognized items. Our results differ from (24) in that our average rate of hippocampal ripples is higher (Table 3, *allRipples*: 13.35/min, *spindleRipples*: 1.14/min), which can be explained by the different detection methods used (amplitude thresholding vs. multivariate analysis). Our results slightly differ with (24) in that the frequency range of the detected ripples was circumscribed to a broader frequency range spanning between 88 and 144 Hz for the *isolRipples*, and 82 to 165 Hz for the *iesRipples*. An important agreement between our results and those from (24) is that hippocampal ripples, when undifferentiated (i.e., *allRipples*), do not present a correlation with performance improvement (measured by the difference in performances obtained pre-sleep and post-sleep).

The strong correlation shown by the *spindleRipples* with the performance improvement provides further evidence for their involvement in memory consolidation processes, moreover, these findings may contribute to the separation of physiological and non-physiological high frequency oscillations in the human hippocampus.

### Limitations

This study presents a grand average of the Ripple activity during cognitive load and does not look into more local phenomena which could arise at specific time points, e.g., the *isolRipples'* activity dynamics at specific time intervals after cue-presentation.

The selection of *spindleRipples* was based on mere co-occurrence, however previous research has shown that Ripples strongly cluster around the troughs of the sleep spindles (15, 25, 26). A selection of *spindleRipples* while considering their clustering around the spindle trough could provide a more depurated sub-set of Ripples promoting the memory-consolidation mechanism.

## Conclusions

In conclusion, the proposed method for the differentiation of physiological and pathological Ripples could help to understand the neural processes that allow the brain to execute cognitive functions such as spatial navigation and may also help to identify specific forms of ripples as biomarkers of epileptogenicity and ictogenicity. We also presented evidence supporting the role of sleep spindle-coincident ripples in memory consolidation processes, which may contribute to better understand the neural interactions allowing the storage of newly acquired information in the brain.

## Data Availability Statement

The original contributions presented in the study are included in the article/supplementary material, further inquiries can be directed to the corresponding author/s.

## Ethics Statement

The studies involving human participants were reviewed and approved by Ethics Committee of the Freiburg University Medical Center. The patients/participants provided their written informed consent to participate in this study.

## Author Contributions

DL-P and LK conducted the navigation tasks with the patients, analyzed the data, and co-wrote the manuscript. AB conducted parts of the navigation tasks with the patients and provided assistance with the EEG measurements. MD provided assistance with the EEG measurements and co-wrote the manuscript. AT helped with the data analysis. AS-B co-wrote the manuscript. All authors contributed to the article and approved the submitted version.

## Conflict of Interest

The authors declare that the research was conducted in the absence of any commercial or financial relationships that could be construed as a potential conflict of interest.
